# Dampening prey cycle overrides the impact of climate change on predator population dynamics: a long-term demographic study on tawny owls

**DOI:** 10.1111/gcb.12546

**Published:** 2014-03-14

**Authors:** Alexandre Millon, Steve J Petty, Brian Little, Olivier Gimenez, Thomas Cornulier, Xavier Lambin

**Affiliations:** 1 Institut Méditerranéen de Biodiversité et d'Ecologie marine et continentale (IMBE), Aix-Marseille Université, UMR CNRS IRD Avignon UniversitéTechnopôle Arbois-Méditerranée Bât. Villemin – BP 80, Aix-en-Provence Cedex 04, F-13545, France; 2 School of Biological Sciences, University of AberdeenTillydrone Avenue, Zoology BuildingUniversity of AberdeenAberdeen, AB24 2TZ, UK; 3 Centre for Human and Ecological Sciences, Forest Research, Northern Research StationRoslin, Midlothian, EH25 9SY, UK; 4 Northumberland Ringing Group37 Stella Hall Drive, Blaydon, Tyne & Wear, NE21 4LE, UK; 5 Centre d'Ecologie Fonctionnelle et Evolutive, CNRSUMR 5175, campus CNRS, 1919 Route de Mende, Montpellier Cedex 5, 34293, France

**Keywords:** demographic rates, functional response, North Atlantic oscillation, population viability analysis, prey cycle, stochastic population dynamics, trophic interactions

## Abstract

Predicting the dynamics of animal populations with different life histories requires careful understanding of demographic responses to multifaceted aspects of global changes, such as climate and trophic interactions. Continent-scale dampening of vole population cycles, keystone herbivores in many ecosystems, has been recently documented across Europe. However, its impact on guilds of vole-eating predators remains unknown. To quantify this impact, we used a 27-year study of an avian predator (tawny owl) and its main prey (field vole) collected in Kielder Forest (UK) where vole dynamics shifted from a high- to a low-amplitude fluctuation regime in the mid-1990s. We measured the functional responses of four demographic rates to changes in prey dynamics and winter climate, characterized by wintertime North Atlantic Oscillation (wNAO). First-year and adult survival were positively affected by vole density in autumn but relatively insensitive to wNAO. The probability of breeding and number of fledglings were higher in years with high spring vole densities and negative wNAO (i.e. colder and drier winters). These functional responses were incorporated into a stochastic population model. The size of the predator population was projected under scenarios combining prey dynamics and winter climate to test whether climate buffers or alternatively magnifies the impact of changes in prey dynamics. We found the observed dampening vole cycles, characterized by low spring densities, drastically reduced the breeding probability of predators. Our results illustrate that (i) change in trophic interactions can override direct climate change effect; and (ii) the demographic resilience entailed by longevity and the occurrence of a floater stage may be insufficient to buffer hypothesized environmental changes. Ultimately, dampened prey cycles would drive our owl local population towards extinction, with winter climate regimes only altering persistence time. These results suggest that other vole-eating predators are likely to be threatened by dampening vole cycles throughout Europe.

## Introduction

Understanding how environmental variation affects the size, persistence and growth rate of natural populations with different life histories is of paramount importance in an increasingly variable world (Lande *et al*., 2003; Boyce *et al*., 2006; Jonzén *et al*., 2010). Indeed, in recent years, evidence has emerged of substantial change in ecosystem processes due to climate change and direct human impact (Tilman *et al*., 2001; Parmesan, 2006). A key endeavour of population ecologists is to unravel the interaction between density and environmental stochasticity, the latter being primarily related to changing climate (Coulson *et al*., 2001; Morris *et al*., 2008; Sæther & Engen, 2010). However, other components of environmental stochasticity besides climate might be also at work, and deserve attention.

Changes in trophic interactions as a consequence of global change, such as in predator-prey systems, may lead to a cascading re-organization of ecosystems (Post *et al*., 1999; Both *et al*., 2009; Estes *et al*., 2011). One of the most spectacular changes in ecosystem dynamics is the dampening of the multi-annual cycles of herbivores (e.g., rodents, lagomorphs, grouse and moths). The amplitude of such cycles dampened nearly simultaneously in a variety of ecosystems over much of Europe in the 1990s (Ims *et al*., 2008; Cornulier *et al*., 2013). Because many such herbivores are keystone species, changes in cyclic dynamics have the potential to disrupt ecosystems, e.g., by forcing predators to prey upon alternative prey species (Kausrud *et al*., 2008; Nolet *et al*., 2013). Pulses of prey productivity in peak years of cyclic systems often translate into enhanced reproduction and survival for many predators (Karell *et al*., 2009; Millon *et al*., 2010; Schmidt *et al*., 2012). Although evidence for declines in vole-eating predators has emerged (Hörnfeldt *et al*., 2005; Millon & Bretagnolle, 2008), a thorough analysis of the impact of dampening vole cycles on predators is required so as to decipher the demographic processes at work.

Two recent studies explored the consequences of changes in lemming dynamics on arctic fox (*Vulpes lagopus*; Henden *et al*., 2008) and long-tailed skua (*Stercorarius longicaudus*; Barraquand *et al*., 2013). Because demographic data for these two species were sparse, mostly hypothetical functional relationships linking predator demographic rates to prey abundance were used in population models. Despite their distinct positions along the slow-fast gradient of life history, the dynamics of both predators were similarly sensitive to changes in average lemming density and temporal variance, while remaining largely unaffected by the cyclic nature of the prey dynamics. This corroborates recent findings on population dynamics of a long-lived bird primarily affected by winter climate and suggests population dynamics might be less affected by the colour of environmental noise than previously thought (van de Pol *et al*., 2011). More generally, one expects the sensitivity of population growth rate to changes in prey density to depend on the shape of the predator's functional response (Jonzén *et al*., 2010; Barraquand *et al*., 2013). Strategies of reproductive investment according to environmental variability are indeed under strong selection pressure (Sæther *et al*., 2004). Therefore, calibrating functional responses for an ensemble of demographic rates to changes in resource availability and climate is central to predict reliable population responses to scenarios of future environmental changes. However, such mechanistic models explicitly accounting for the multifaceted effects of global changes remain rare (Brook *et al*., 2009; van de Pol *et al*., 2010; Jenouvrier, 2013).

Long-term demographic datasets spanning a large range of environmental conditions are invaluable in this context (Jenouvrier, 2013). Here, we used 27 years of longitudinal data on tawny owls (*Strix aluco*) and their field vole (*Microtus agrestis*) prey to elucidate the interaction between prey density and climate on predator demography and its response to future environmental changes. In Kielder Forest (northern England), this predator-prey system has experienced a profound shift in prey dynamics from high- to low-amplitude cycles in the mid-1990s, even though it remains cyclic (Cornulier *et al*., 2013). We first characterized the functional responses of first-year and adult survival, breeding probability and fecundity of the predator to season-specific prey densities. Second, we investigated how predator demographic rates responded to winter climate in addition to prey density. Lastly, we translated environmentally driven variations in predator demographic rates into a stochastic population model. We then predicted predator population size according to a simulation experiment crossing scenarios of change in prey dynamics with a range of winter climate conditions. Our aim was to test whether climate may buffer or alternatively magnify the impact of changes in prey dynamics.

## Material and methods

### Study species and site

Since 1979, we have monitored a population of tawny owl breeding in nest boxes in the centre of Kielder Forest (176 km², 55°13′ N - 2°33′ W, Petty, 1992). Owls from this population are relatively long-lived, site-faithful and are able to breed when 1-year old (Millon *et al*., 2010). This nocturnal predator preys upon small vertebrates, especially field voles taken from open grassy areas formed by clear-cuts within an intensively managed spruce forest (Petty *et al*., 2000). Field voles represented on average 62% of the prey (range: 35–75%; 48% of biomass) brought to the nest by tawny owls during the breeding season (March–June; Petty, 1999). Few owl pairs were able to breed when the proportion of voles in their diet fell below 50%. Thus, in Kielder Forest, the tawny owl could be categorized as a specialist predator due to a relative dearth of alternative prey.

Field vole densities were derived from sign index surveys (presence/absence of fresh feeding remains) conducted in spring and autumn on 13–26 sites since autumn 1984 (details in Lambin *et al*., 2000). Time series of field vole densities in Kielder Forest, as in much of Europe, show a strong cyclic signal over a 3–4 years period, with more than an order of magnitude in amplitude within individual sampling sites (20–700 voles ha^−1^).

Tawny owl nest boxes were visited at least three times between March and June to record occupancy, whether eggs were laid, and to count and ring chicks before fledging. Almost all breeding females were caught annually during 1984–2010 (> 90%; Millon *et al*., 2011). Birds in this population do not roost in nest boxes thus precluding the capture of floaters and nonbreeding territorial birds. When breeding did not occur in a territory, we assessed whether this territory was occupied by checking for the appearance of a nest cup during the breeding season, as territorial tawny owls leave a clearly detectable scrape even when failing to lay. In contrast to tawny owl, other species that occasionally occupy nest boxes in this area typically add materials to the nest box such that we are confident that this method truly relates to tawny owl occupancy. Data on nest scrapes were missing for 5 years. Given virtually all chicks about to fledge have been ringed in Kielder Forest since 1981, we assumed that nonringed birds caught as breeders from 1985 onwards were immigrant.

We assessed the severity of winter conditions in Kielder Forest using the wintertime (December through March) North Atlantic Oscillation index based on the difference of normalized sea level pressure between Portugal and Iceland (hereafter wNAO; http://climatedataguide.ucar.edu/guidance/hurrell-north-atlantic-oscillation-nao-index-station-based). This index often better explains variation in many ecological processes than covariates such as monthly temperature or precipitation, as it integrates them over a longer and more relevant time scale (Hallett *et al*., 2004).

### Modelling functional responses of predator demographic rates to prey density and winter climate

Vole densities strongly influence the demography of tawny owls, especially first-year survival and reproduction (Karell *et al*., 2009; Millon *et al*., 2010, 2011). However, climate may also shape demographic rates (Lehikoinen *et al*., 2011). For each demographic rate, we considered a set of five hypothetical models including (i) vole density, (ii) wNAO, (iii) vole density + wNAO, (iv) vole density × wNAO and (v) a null model assuming a constant demographic rate. The model with an interaction between prey density and winter climate specifically tested the hypothesis that climate might buffer (or magnify according to the sign of the interaction) the effects of prey density on demographic rates. We checked whether a log-transformation of vole density improved the model fit for each demographic rate and used the most appropriate covariate for the model selection process. We also checked that the density of territorial owls had no direct influence on demographic rates (results not shown; see below for the way we modelled territoriality).

Age-specific survival of females was estimated through capture/recapture analyses conducted in E-Surge (Choquet *et al*., 2009). The first data set included birds ringed as chicks and recorded recapture histories of 26 cohorts born between 1984 and 2009 (*N* = 1019 chicks; recaptures recorded until 2010; see Appendix S1 for sex assignment). We tested for a possible nonlinear relationship between prey density and first-year survival by specifying a nonparametric flexible relationship using penalized splines (Gimenez *et al*., 2006). First-year survival is apparent survival, i.e., it considers birds having left the study area as dead. A distinct data set pooling locally recruited female chicks with females first marked when breeding was assembled to estimate adult survival (*N* = 248). In contrast to first-year survival, adult survival is close to true survival, as movement of adults following recruitment was rare (*N* = 26 out of 357 female/years) and involved movement to adjacent territories in all cases.

Fecundity was decomposed into two distinct demographic processes: (i) the probability of breeding was estimated as the ratio between the number of pairs having laid at least one egg (breeding pair) and the total number of territory occupied (whether breeding occurred or not, *N* = 1182), and (ii) the number of offspring fledged per breeding pair (*N* = 977). These two response variables were fitted using generalized linear models with binomial and multinomial distributions of error, respectively (see Appendix S2 regarding the choice of the multinomial distribution). As with survival, the effect of vole density and winter climate was tested through the comparison of the same five competing models.

For each demographic rate, the most parsimonious functional relationship was identified based on Akaike's Information Criterion corrected for small sample size (AICc; Burnham & Anderson, 2002) and incorporated into the demographic model.

### Stochastic demographic model

**Fig. 1:**
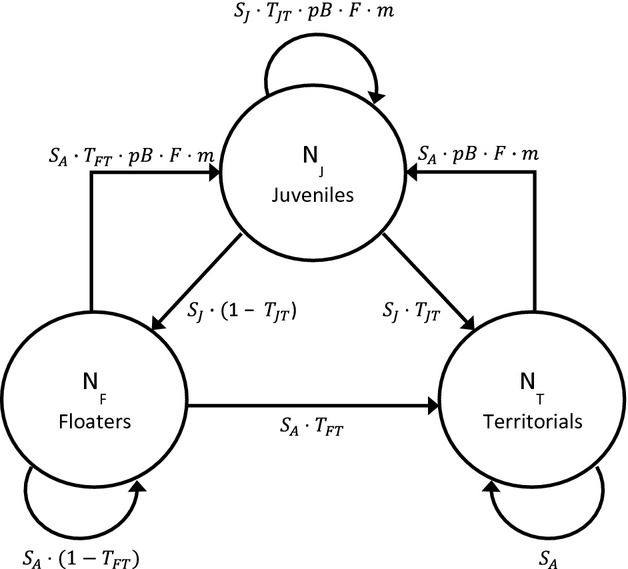
Schematic view of the three-stage life cycle (juveniles *N*_J_, floaters *N*_F_, territorials *N*_T_) describing the demography of female tawny owls. See Methods for abbreviations.

We modelled tawny owl life history as a three-stage annual life-cycle accounting for juveniles (*N*_J_), floaters (*N*_F_) and territorial birds (*N*_T_, Fig. 1). The population model monitored females only, according to the following matrix projection (Caswell, 2001):


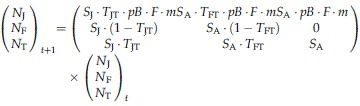


This model tracks stage-specific population sizes just after reproduction (postbreeding census) and therefore involves four sequential demographic processes (survival, recruitment, reproduction and immigration).

1. Birds survive with age-specific probabilities (*S*_J_ & *S*_A_ for first-year and adult survival, respectively). For the sake of simplicity, we did not incorporate the senescence observed in survival for this species (Millon *et al*., 2011).
2. Birds move between stages. We accounted for the territorial behaviour of tawny owls by setting an arbitrary maximum number of territories (*N*_Tmax_ = 100). Transition probabilities (T) were set according to territory availability and were therefore density dependent. Hence, the probability that a floater becomes territorial was dependent upon the number of vacancies created by mortality of territorial birds. We assumed floaters had priority over juvenile birds in acquiring vacant territories so that the probability of a floater gaining a territory was:
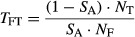
  If some territories remained vacant following the recruitment of floaters, then juveniles could access territories with probability: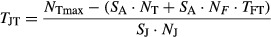  When the number of potential recruits was less than the number of territory vacancies, recruitment rates *T*_JT_ and *T*_FT_ were set at 1. Once *N*_Tmax_ was reached, surplus juveniles became floaters, and surplus floaters remained in that stage. We assumed no territory take-over (i.e*. T*_TF_ = 0, territory takeover being females is rare in Kielder Forest [1%, *N* = 180]).
3. Once territorial, birds reproduce according to a probability of breeding (*pB*). Breeding females raise a number of female offspring (*F*) set as half the average number of fledglings per breeding female (assuming an even sex ratio).
4. Immigration contributes to the number of juveniles according to a *per capita* immigration rate *m*. We estimated *m* = 1.728 from the ratio between immigrant (*N* = 91) and locally born female recruits (*N* = 125) in Kielder Forest between 1985 and 2010. Implementing immigration in such a way assumes synchronous fluctuations of prey dynamics (and winter climate) over large-spatial scales and therefore imposed the number of immigrants to covary with fecundity.

Demographic stochasticity was accounted for in the demographic model by drawing the probabilistic application of demographic rates to each individual (binomial distributions for survival, probability of breeding and sex assignment, and a multinomial distribution for number of fledglings). Incorporating density dependence in the population model (ceiling in the number of territory available making transitions between stages density-dependent; see van de Pol *et al*., 2010) renders population growth rate *λ* density-dependent as well, and thus largely uninformative (Caswell, 2001). Therefore, we used the median projected number of territorial females after 250 years, instead of *λ*, for describing population dynamics. The initial population vector was fixed as follows: *N*_T_ = 100, *N*_F_ = 20, *N*_J_ = 75. Simulation results were remarkably insensitive to the initial number of floaters (tested range: 10 ≤ *N*_*F*_ ≤ 50).

### Building scenarios of change for prey dynamics and winter climate

We aimed to test whether variation in winter climate has the potential to buffer the negative effects of low-amplitude vole cycles or to counteract the positive effects of high-amplitude vole cycles. To do so, we modelled owl population dynamics preying upon simulated vole population time series, allowing vole cycles amplitude and climate regime to vary within the range observed according to a factorial design.

**Fig. 2:**
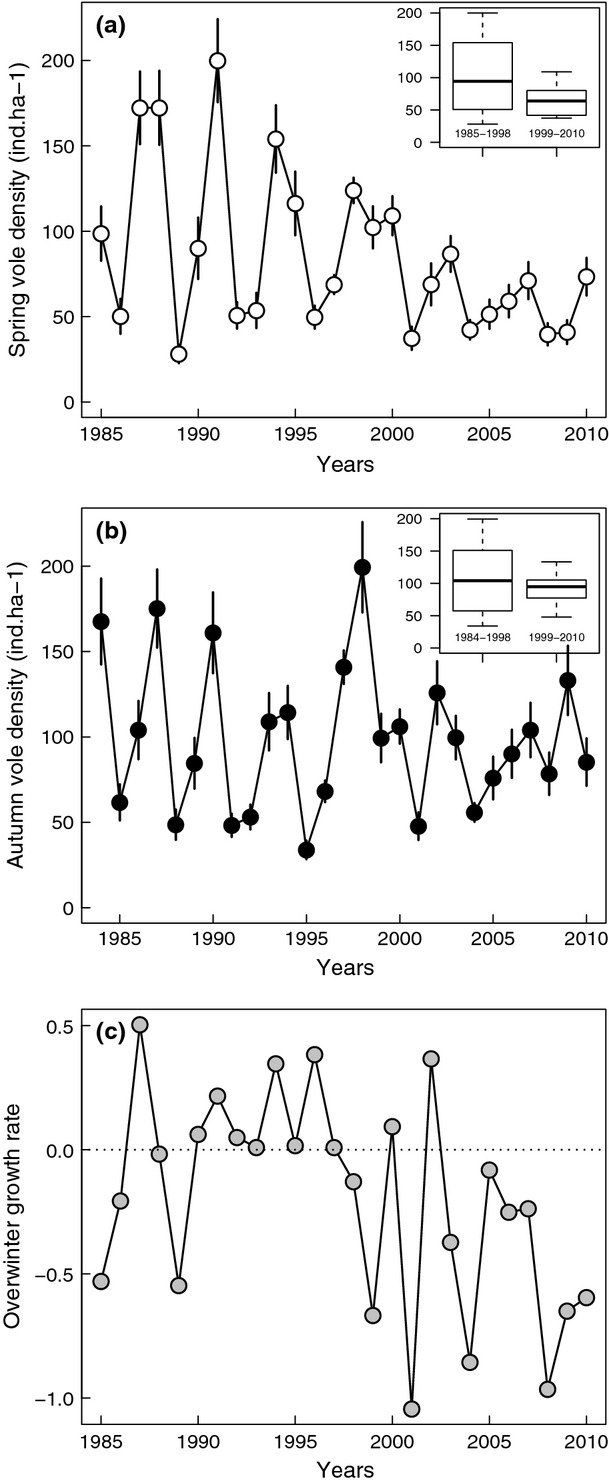
Time series of field vole densities (mean ± SE) measured in spring (a) or autumn (b), and of overwinter population growth (c) in Kielder Forest. Inserted panels in (a) and (b) contrast field vole densities between the high- and low-amplitude periods (1984(5)–1998 and 1999–2010, respectively).

Changes in vole dynamics across Europe appear to be driven by a change in basal winter population growth rates rather than a change in density-dependent regulation (Cornulier *et al*., 2013). In Kielder Forest as elsewhere in Europe, vole dynamics shifted from high- to low-amplitude cyclic regime. The decrease in winter growth rate of vole populations translated into lower spring vole densities, whereas mean autumn density remained roughly unchanged (but variance decreased, Fig. 2a–c). We used the model and parameter estimates of Cornulier *et al*. (2013) to simulate season-specific vole densities with varying amplitude regimes. Specifically, we varied winter population growth rate (18 increments of 0.1 on log scale), while keeping other parameters constant at their 1984–2010 average. This produced synthetic vole time series that captured the observed variation in spring and autumn densities during the high and low-amplitude periods while keeping key features of the real population trajectories (Appendix S3). For illustrative purpose, we defined two contrasted periods with high- (1984–1998) and low-amplitude (1999–2010) vole dynamics.

**Fig. 3:**
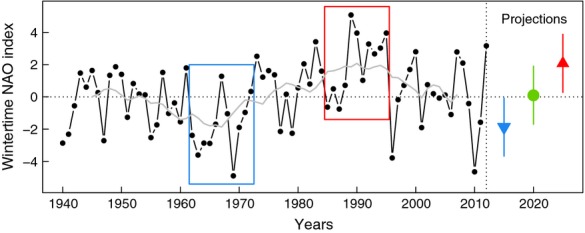
Time series of wintertime NAO. Decadal variability produced a shift from a negative period in 1962–1972 (mean (± 1SD) = −1.87 ± 1.75), characterized by cold and dry winter climate in north-western Europe, to a positive period in 1985–1995 (2.08 ± 1.99) characterized by warmer but wetter conditions in winter (grey line: moving-average using an 11-year window). The three winter climate regimes defined for the scenarios are indicated by the coloured symbols on the right [negative with blue triangle down: mean = −1.87; positive with red triangle up: 2.08; intermediate with green circle: 0.105 (average between positive and negative periods); standard deviations were set equal (SD = 1.8) for the three simulated regimes, so as to make them to differ by average values only (draw from a normal distribution assuming no autocorrelation)].

The North Atlantic Oscillation index correlates with low-frequency (decadal) changes in European climate and a regime shift occurred from mostly negative (around 1967, cold and dry winters) to mostly positive wNAO index (around 1990, wet and mild winters; Visbeck *et al*., 2001). In northern England (Windermere, 80 km SSW of Kielder Forest), wNAO was strongly and positively correlated with air temperature and precipitation (rain) in winter, but negatively correlated with the duration of the snow cover (1964–1999; George *et al*., 2004). For our analysis, we created three distinct regimes of wNAO: a negative and a positive regime based on observations (11-year window), plus an intermediate regime such as the one observed in the 2000s (Fig. 3). Our study period mostly coincided with a positive regime of wNAO (mean ± 1SD = 0.85 ± 2.31, 1st–3rd quartiles: [−0.35, 2.76]; Fig. 3). There was no direct relationship between wNAO and vole density in spring (*r*_p_ = 0.08, *P* = 0.70, *N* = 26) nor overwinter population growth (*r*_p_ = 0.14, *P* = 0.48, *N* = 26).

We generated 10,000 simulations of seasonal prey density over 250 years for each combination of prey dynamics and wNAO regimes (18 × 3 combinations). At each time-step, vole densities for spring and autumn were taken from the simulated prey dynamics, and the wNAO index was drawn from a normal distribution defined for each of the three winter regimes. Prey and wNAO were then used to calculate the annual value for each predator's demographic rate based on specific functional relationships. Residual temporal variance in demographic rates was calculated as the difference between observed yearly averages and predicted values from the functional relationships. Such uncertainty in parameter estimation was included in the population model by drawing at each time step a vector of residual temporal variance that maintained the covariance among demographic rates.

### Perturbation analyses

We conducted a life table response experiment so as to compare the relative impact of an environmental perturbation occurring in prey density or winter climate. We chose vole dynamics producing an average vole density in spring matching the observed values in Kielder Forest over the study period (∼85 vole ha^−1^) and intermediate wNAO regime as reference conditions. Vole density and wNAO were altered by adding to simulated values 5% of the observed difference in averages between low- and high-amplitude periods (1.84 vole ha^−1^) and between negative and positive wNAO regimes (0.198), respectively. We measured the response of owl population in terms of median number of territorial females (*N*_T_) after 250 years.

Analyses and modelling were conducted in R 2.11.1 (R Development Core Team, 2010).

## Results

### Functional responses of predator demographic rates to prey density and winter climate

**Fig. 4:**
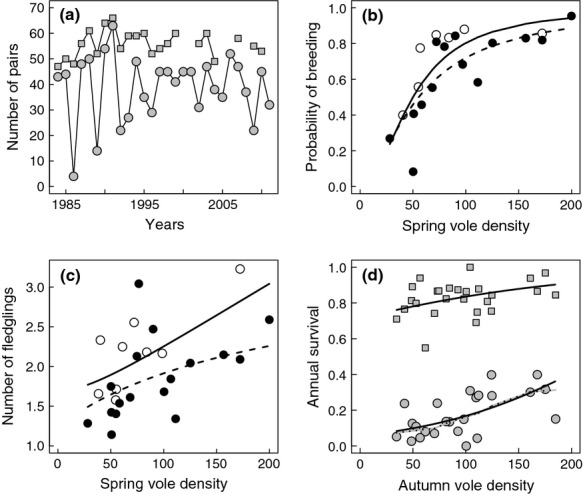
(a) Number of tawny owl pairs in Kielder Forest (squares: territorial pairs, circles: breeding pairs). No data available for territorial pairs in 2000–2001, 2005–2006 and 2008. Functional responses of (b) probability of breeding, (c) number of fledgling per breeding pair and (d) age-specific survival (circles: first-year, squares: adult) to season-specific vole density. Black and white symbols are for years with positive and negative wNAO index, respectively, if the best model included wNAO (see Table 1). Lines are predicted values taken from the best model (solid and dashed lines for 1st and 3rd quartiles of wNAO, respectively). The dotted line for first-year survival was drawn with the results from the non-parametric spline method.

The number of territorial pairs oscillated between 47 and 66 with a slight increase until 1991 followed by a slight decline (Fig. 4a). The number of breeding pairs varied much more, ranging from 4 to 63, although the amplitude of this variation decreased during the low-amplitude period (range: 30–51). Breeding probability was strongly dependent upon vole density in spring, with typically 80% of territorial pairs breeding in years when vole density exceeded 70–75 voles ha^−1^ and a sharp decline occurring below this threshold (Fig. 4b). As wNAO decreased, the breeding probability of tawny owls increased, although this effect tended to vanish at very low vole density (Table 1). Given 100 voles ha^−1^ in spring and a low wNAO (1st quartile), the probability of breeding increased by ca. 0.08 compared to a high wNAO (3rd quartile), which is equivalent to a change of 25–35 voles ha^−1^. The best model explained almost two-thirds of the deviance (*R*² = 63.7%).

**Table 1:** Selection of models investigating the functional response of four demographic rates to vole density and winter climate. The number of parameters (np), the AICc difference between the current model and the most parsimonious one (ΔAICc), AICc weights (wAICc) and proportion of deviance explained (*R*²) are shown. Models with the strongest support are indicated in bold

Demographic rate and model	np	ΔAICc	wAICc	*R²*	Equation
Probability of breeding					
Null	1	180.5	0	–	
Log(Vole in spring)	2	8.9	0.01	58.9	
wNAO	2	155.5	0	5.0	
Log(Vole) + wNAO	3	1.4	0.33	62.4	
**Log(Vole)** × **wNAO**	**4**	**0**	**0.66**	**63.7**	logit(pB) = −7.777 + 1.983 × log(vole_spring_) + 0.478 × wNAO–0.136 × log(vole_spring_) × wNAO
Number of fledglings					
Null	5	128.2	0	–	
Log(Vole in spring)	10	59.6	0	16.6	
wNAO	10	103.6	0	7.3	
Log(Vole) + wNAO	15	2.47	0.23	30.8	
**Log(Vole)** × **wNAO**	**20**	**0**	**0.77**	**33.5**	See Appendix 2
1st-year survival					
Null	30	10.0	0	–	
**Vole in autumn**	**31**	**0**	**0.66**	**45.6**	logit(S_J_) = −1.640 + 0.512 × vole_autumn_
wNAO	31	11.3	0	3.0	
Vole + wNAO	32	2.0	0.24	45.9	
Vole × wNAO	33	3.7	0.10	47.4	
Adult survival					
Null	32	5.4	0.03	–	
**Vole in autumn**	**33**	**0**	**0.49**	**22.4**	logit(S_A_) = 1.602 + 0.299 × vole_autumn_
wNAO	33	6.7	0.02	2.4	
Vole + wNAO	34	0.9	0.32	26.1	
Vole × wNAO	35	2.5	0.14	27.7	

Average number of fledglings per breeding pair varied among years from 1.9 to 3.2 (Fig. 4c, Appendix S2). As with breeding probability, the best model retained the interaction between vole density and wNAO, resulting in a steeper positive relationship between the number of fledglings and spring vole density in years with negative wNAO. Given 100 voles ha^−1^, a low wNAO index increased the number of fledglings by about 0.25–0.5 chicks compared with a high wNAO index, which is equivalent to a change of 30–60 voles ha^−1^. In contrast to the probability of breeding, wNAO significantly increased the proportion of deviance explained compared to a vole-only model (from 16.6 to 33.5%; Table 1). Reduced number of fledglings following wet and mild winters (positive wNAO) primarily arose from a reduction in clutch size (rather than more severe brood reduction; Appendix S4).

First-year apparent survival varied from 0 to 0.40, with 95% of yearly estimates comprised between 0.08 and 0.28 (Fig. 4d). Variation was to a large extent explained by vole density in autumn (*R* = 45.6%, Anodev test: *P* < 0.001; Table 1). There was no evidence for nonlinearity in this relationship (fitting curve from the nonparametric spline method being very similar to the parametric one, Fig. 4d). Adult female survival ranged between 0.55 and 1 (95% interval: 0.77–0.88), and variation was also positively correlated with vole density in autumn, though to a lesser extent compared to first-year survival (*R*² = 22.4%, Anodev test: *P* = 0.01; Table 1). Winter climate did not significantly impact age-specific survival of tawny owls.

### Effect of prey dynamics and winter climate on predator dynamics

**Fig. 5:**
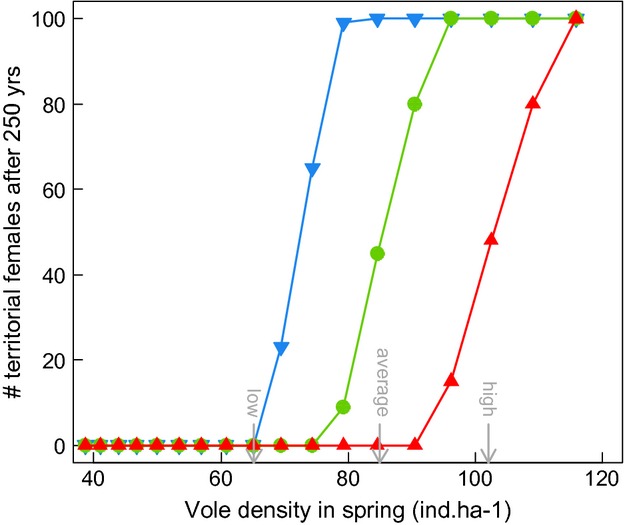
Projected median number of territorial females (*N*_T_) after 250 years according to mean spring vole densities as simulated by autoregressive models (each point differs by 0.1 unit of vole winter population growth). The three different curves indicate simulations performed under positive (red, triangle up), intermediate (green, circle) and negative (blue, triangle down) wNAO regimes. Arrows indicate spring vole densities observed in Kielder Forest during low- and high-amplitude vole cycle periods, and averaged over the whole period.

**Fig. 6:**
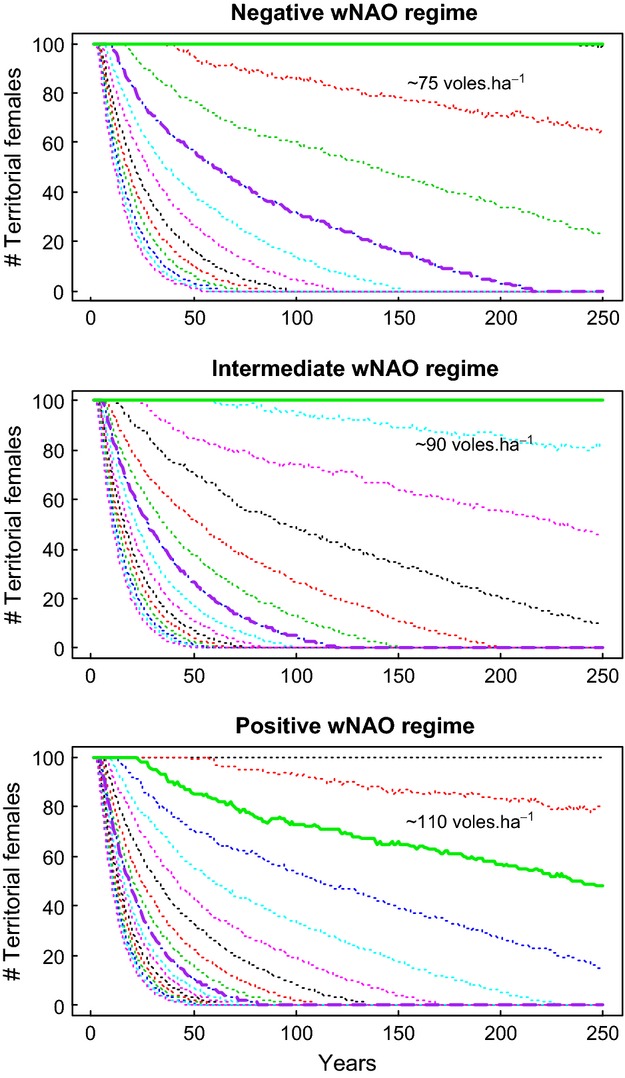
Projected median number of territorial females (*N*_T_) over 250 years according to simulated spring vole densities (dotted lines; same simulations as in Fig. 5). Solid and dashed bold lines show projections with vole dynamics matching spring vole densities observed during high- (green) and low-amplitude (purple) vole cycle periods, respectively. Average spring vole density is indicated for the first strongly declining simulation.

Projected population dynamics of tawny owls in Kielder Forest were strongly affected by prey dynamics. The median population size (of territorial females) declined sharply when average vole spring density fell below 75–105 voles ha^−1^ (Figs 6). Under high-amplitude vole dynamics as seen in 1984–1998, the median projection of territorial females remained at carrying capacity, i.e., all territories were occupied, except under prolonged period of adverse winter (wNAO positive). Simulating low-amplitude vole dynamics as observed in 1999–2010 precipitated owl population extinction, irrespectively of winter climate. Time of persistence increased from 82 to 215 years, when shifting from positive to negative wNAO regime (Fig. 6). Confidence intervals were, however, relatively wide (Appendix S5). The effect of a shift from positive to negative wNAO period on owl population dynamics was roughly equivalent to a an increase in 25–30 voles ha^−1^ in spring (Fig. 5).

Life table response experiments highlighted the stronger absolute impact of spring vole density compared to wNAO (Table 2). Increasing vole density in spring by adding an amount equivalent to 5% of the observed variation in Kielder Forest increased *N*_T_ by 38%. This increase was mainly mediated by improved probability of breeding. Increasing wNAO by adding an amount equivalent to 5% of the variation between average values observed between negative and positive regimes decreased *N*_T_ by 22%. Most of this perturbation was, however, mediated by the average number of fledglings.

**Table 2:** Life table response experiment results for the number of territorial females (*N*_T_) after 250 years. An intermediate wNAO regime and vole dynamics producing an average vole density in spring matching the observed average value in Kielder Forest (∼85 ind.ha^−1^) were used as reference environmental conditions (projecting *N*_T_ = 45 after 250 years). Altered conditions were sequentially applied to the probability of breeding, number of fledglings, and to both demographic rates simultaneously. Age-specific survival rates were ignored as they are related to autumn vole density only

	Δ*N*_T_ after Perturbation	% absolute perturbation
Perturbation of vole density in spring		
Probability of breeding	12	0.27
Number of fledglings	5	0.11
Both	17	0.38
Perturbation of wNAO		
Probability of breeding	−2	0.04
Number of fledglings	−6	0.13
Both	−10	0.22

## Discussion

Using stochastic demographic modelling, we showed that a tawny owl population from northern England is highly vulnerable to dampening prey cycle observed there and throughout Europe (Cornulier *et al*., 2013). High-amplitude vole cycles as observed in the 1980s would maintain number of territorial owls near carrying capacity, largely irrespective of winter climate. In contrast, prolonged dampening periods of vole cycles could drive our study population to extinction, with change in winter climate only affecting time to extinction. Overall, our study showed that a relatively long-lived species may suffer more from decreasing abundance of an autocorrelated resource rather than from the influence of climate.

### Demographic functional responses to changing resource dynamics and climate

A common feature of vole dynamics in Europe is reduced spring vole density, while density in autumn remains less affected (Cornulier *et al*., 2013; Fig. 2). This was captured by our simulated vole time series, such that the projected collapse of the owl population can unambiguously be ascribed to the reduced probability of breeding and to a lesser extent to the lower number of fledglings, rather than to survival. Indeed, we found that age-specific survival rates were positively and linearly linked to vole density in autumn (and not spring), while wNAO had little effect. In contrast, the probability of breeding and especially the number of fledglings were related to the dual effects of spring vole density and winter climate. Tawny owls start breeding at the end of winter (95% start incubation between March 1st and April 22nd). Therefore, birds might struggle to reach physiological condition required for breeding when facing wet and mild winters. Pairs breeding in those harsh years also laid fewer eggs and consequently raised fewer chicks.

We performed a perturbation analysis to disentangle the relative contribution to time to extinction of demographic rates altered by changes in spring vole density and wNAO. Whereas the effect of winter climate was mainly mediated by the average number of fledglings, the probability of breeding accounted for most of the effect induced by spring vole density. Overall, the demography of tawny owl in Kielder Forest was primarily affected by variation in season-specific prey density and less so by winter climate (the vole effect was 1.7 times greater than the climate effect; Table 2). It can be argued that the use of specific climatic variables instead of wNAO could have led to a slight increase of the climate effect on owl demographic rates. Nevertheless, the relatively weak impact of winter climate on demographic rates is not surprising for a population relatively far from the species' range margins, and we expect marginal populations to be more affected by winter climate. The impact of winter climate on demographic rates is indeed expected to vary according to geographic locations and across species (Grøtan *et al*., 2009). Population dynamics have been shown to vary among populations: cold and wet weather conditions reduced reproductive output in one spotted owl (*Strix occidentalis*) population, while they improved it in another (Peery *et al*., 2012). Furthermore, breeding parameters of four owl species showed contrasted responses to winter temperature or snow depth in Finland (Lehikoinen *et al*., 2011). Interestingly, future winter climatic conditions are predicted to benefit Oystercatchers (*Haematopus ostralegus*) through improved winter survival and despite a negative effect on resource availability affecting reproductive success (van de Pol *et al*., 2010). Sensitivity of demographic processes to environmental conditions can thus greatly vary and complicate generalization among populations and across species about the impacts of global change (Sæther & Engen, 2010).

Only a handful of studies have assessed the future of bird populations in the face of global change by integrating functional responses into a comprehensive population model (Jenouvrier *et al*., 2009; van de Pol *et al*., 2010; Hunter *et al*., 2010; review in Jenouvrier, 2013). Climate is often assumed as the main component of environmental stochasticity by acting either directly on demographic rates (e.g. winter temperature altering survival) and/or indirectly through impacts on resources (e.g. food shortage affecting reproduction; Sæther & Engen, 2010). These different pathways can buffer or alternatively magnify the impact of environmental changes. Even if some populations may gain direct benefits from climate change, such benefits may be swamped by reduced resource availability. Therefore, predicting the fate of populations facing changing environments requires a careful integration of the ecological processes involved, and of trophic interactions in particular (Martin, 2007; van de Pol *et al*., 2010; Peers *et al*., 2014).

Here, we considered the impact of prey density and climate on tawny owls as independent processes, as we did not detect direct correlation between vole dynamics and wNAO. An important question remains though, as to whether climate change can affect vole dynamics, as documented for lemmings (Ims *et al*., 2011). It has been suggested that dampened cycles may arise following warmer and wetter winters and earlier onsets of spring (Bierman *et al*., 2006), although this remains to be properly tested over a range of locations. If wetter winters lengthened the period of dampened cycles in the future this would amount to the worst-case scenario for predators of cyclic voles such as the tawny owl in Kielder Forest.

### In what circumstance might dampened prey cycles drive predator populations to extinction?

Based on a large compilation of vole time series, Cornulier *et al*. (2013) showed that cycle amplitude dampening occurred across a wide range of ecosystems in Europe. Therefore, our results do not portend well for vole-eating predators in Europe (e.g. nocturnal and diurnal raptors and mustelids). There is some evidence of large-scale declines in populations of several vole-eating specialist predators with wide distribution such as European kestrel (*Falco tinnunculus*), short-eared owl (*Asio flammeus*), barn owl (*Tyto alba*) and possibly boreal owl (*Aegolius funereus*) (http://www.birdlife.org/datazone, Hörnfeldt *et al*., 2005).

While our simulations assumed constant vole dynamics (though with stochastic annual realizations), nonstationary rodent cyclic dynamics have been documented on a local scale (Henden *et al*., 2009) and alternating periods with low- and high-amplitude vole might occur at a larger scale as well. Then, duration of periods with dampened cycle and the extent of spatial synchrony of this phenomenon would be key parameters for the persistence of vole predators' populations. Predators can buffer shortage of prey through nomadism (tracking of high prey density over large spatial scales), migration (movements during the non-breeding season) or by switching to alternative prey (generalist predation). Longevity also provides some buffer against environmental changes (Morris *et al*., 2008). Barraquand *et al*. (2013) showed that the longevity of skuas (median duration of adult life stage 1/(1–S_A_): 14 years relative to 6 years for tawny owl) enables populations of this species to persist over >10 years with virtually no reproduction, provided the floater-to-breeder ratio is close to one at equilibrium. In contrast to skuas, first-year survival of tawny owls (like other boreal owls) is low and remains tightly linked to prey density during the first winter and beyond. This restricts the production of floaters and hence the population's capacity to cope with long periods of prey shortages. Our data and model results indicated this ratio is constantly much lower than one for tawny owl, and that the floater compartment can be rapidly depleted (Appendix S6; Millon *et al*., 2010).

### Life-history evolution and population dynamics in a stochastic world

Life-history evolution predicts that variability in demographic rates is selected against because of its negative impact of variability on population growth (Gillepsie, 1977). Therefore, we expect a negative relationship between temporal variability and contribution of demographic rates to population growth (Pfister, 1998; Gaillard & Yoccoz, 2003). This view has been recently challenged based on growing evidence that the functional form linking demographic rates to environmental variables is crucial for determining population response to environmental changes. Koons *et al*. (2009) proposed that demographic lability, rather than demographic buffering, may be adaptive for some traits of species living in highly variable environment. Environmental variability can indeed have a positive impact on growth rates of natural populations provided that functional relationships are non-linear and log-convex (Henden *et al*., 2008; Jonzén *et al*., 2010; Barraquand *et al*., 2013). It is still unclear, however, how common this positive effect of environmental variability is (Barraquand & Yoccoz, 2013). Reduced variance but constant mean in autumn prey density are likely to be profitable for tawny owls by improving first-year and adult survival, given their linear relationships with prey density. However, this positive effect is swamped by the reduction in mean spring vole density and the concave relationship linking probability of breeding to prey density.

Evolutionary response may allow populations to cope with future environmental conditions (Lavergne *et al*., 2010). Here, we did not consider any adaptive response of tawny owls that may buffer the negative impact of reduced vole densities. Interestingly, however, tawny owls show polymorphism in their ability to exploit alternative prey when voles are scarce (Roulin *et al*., 2003; Brommer *et al*., 2005). Thus, genotypes with different abilities to cope with a highly unstable resource like voles and a changing climate may coexist. The loss of high-amplitude cycles may change the outcome of selection acting on predatory behaviour by favouring generalist genotypes, for which reproduction is less sensitive to vole density, over specialist genotypes.

In conclusion, by incorporating detailed functional relationships and accounting for different sources of stochasticity, our modelling work sheds light on the consequences of a major change in ecosystem functioning that is on-going throughout Europe. While the deleterious impact of dampening lemming cycles in arctic ecosystems on predators had already been documented (Schmidt *et al*., 2012), our study indicates that the large guild of vole-eating predators of temperate European ecosystems may also be vulnerable to dampening vole cycles.

## Supporting Information

**Appendix S1.** Procedure for sex-specific first-year survival estimates. **Appendix S2.** Modelling the variation in number of fledglings using a multinomial distribution. **Appendix S3.** Procedure for simulating vole time series and comparison with observed data. **Appendix S4.** Functional response of clutch size of tawny owls to spring vole density and wNAO. **Appendix S5.** Confidence intervals for the simulated number of territorial female tawny owls in Kielder Forest according to varying mean vole density in spring. **Appendix S6.** Simulated dynamics of non-territorial tawny owls (floaters) according to varying vole dynamics.
