# Supplementary material for: Dampening prey cycle overrides the impact of climate change on predator population dynamics: a long-term demographic study on tawny owls
Source: Glob Chang Biol. 2014 Mar 14;20(6):1770–81. doi: 10.1111/gcb.12546 (PMC4320692; doi:10.1111/gcb.12546)
Supplement: Supplementary file 1 [file gcb0020-1770-sd1.docx]

**Appendix S1. Procedure for sex-specific first-year survival estimates.**

The chicks of tawny owls cannot be sexed without recourse to molecular techniques. This has been done for all chicks ringed in Kielder Forest (N = 312) over five consecutive years (1994-1998) with contrasted vole densities. Overall, this analysis revealed an even production of females (N = 156) and males (N = 156). Furthermore, sex-ratio at the population level was found to be insensitive to vole densities as it did not depart from parity in any of the years (binomial test conducted for each year separately, all P’s > 0.66). Based on this result, we randomly assigned the sex of birds born in years without molecular sexing and that were never recaptured so as to obtain an even number of ringed male and female chicks in each year (when combining birds having been subsequently recaptured or not). Assuming an even sex-ratio in each year, this method provides unbiased annual estimates of apparent first-year survival for either sex.

**Appendix S2. Modelling the variation in number of fledglings using a multinomial distribution.**

As a consequence of a low variance/mean ratio (1.25/2.09 = 0.60) observed in the data, the Poisson distribution failed to produce accurate predictions for the number of fledglings per breeding pair, i.e. pair having laid at least one egg (see McDonald & White, 2010 for a similar case in California spotted owls). We tested a variety of alternative distributions (Gaussian, log-Normal), and found that the multinomial distribution was the most adequate to predict the number of fledglings. Such a model predicts the relative probability for each brood size observed at fledging (from zero to five; Table S2 & Figure S2).

Reference

McDonald TL, White GC (2010) A comparison of regression models for small counts. *Journal of Wildlife Management*, **74**, 514–521.

Table S2: Coefficients and standard errors from the best multinomial model explaining variation in the number of fledglings (log(Vole)×wNAO; see Table 1 in the manuscript).

| Number of fledglings | Intercept | log(Vole in spring) | wNAO | log(Vole) ×wNAO |
| --- | --- | --- | --- | --- |
| *Coefficients* | |  |  |  |
| 1 | 1.399 | -0.334 | 0.464 | -0.087 |
| 2 | 2.797 | -0.372 | -0.605 | 0.142 |
| 3 | -2.378 | 0.725 | -0.795 | 0.144 |
| 4 | -9.226 | 1.989 | 1.292 | -0.376 |
| 5 | -24.902 | 4.672 | 2.607 | -0.673 |
| *Standard errors* | |  |  |  |
| 1 | 1.481 | 0.345 | 0.598 | 0.142 |
| 2 | 1.155 | 0.268 | 0.523 | 0.123 |
| 3 | 1.196 | 0.274 | 0.610 | 0.141 |
| 4 | 1.602 | 0.352 | 0.885 | 0.203 |
| 5 | 7.785 | 1.533 | 3.781 | 0.785 |

Figure S2: Relative probabilities of brood size at fledging (per breeding pair) according to spring vole density predicted by the best model (log(Vole)×wNAO) under (a) a low wNAO regime (1^st^ quartile, wNAO = -0.35) and (b) a high wNAO regime (3^rd^ quartile, wNAO = 2.76). The width of the grey area is proportional to the probability of observing a given brood size and for a given vole density, the relative probability for each brood size sums to one.

**Appendix S3. Procedure for simulating vole time series and comparison with observed data.**

In Kielder Forest (northern England), as elsewhere in Europe, vole dynamics showed dampening amplitude in cycles characterised mainly by low vole densities in spring, while vole densities in autumn remained relatively unaffected (Cornulier *et al.*, 2013). We used the model in Cornulier *et al.* (2013) to simulate vole population dynamics in Kielder Forest. This model is a seasonal log-linear auto-regressive model for time series. Let *S_t_* and *F_t_* the spring and autumn (fall) log abundances of voles in year *t*. The seasonal growth rates over winter and summer are respectively modelled as:

$S_{t}=a_{0}+\left( 1+a_{1} \right).F_{t-1}+a_{2}. S_{t-1}+ a_{3}. F_{t-2}+ a_{4}. S_{t-2}+ \varepsilon_{t}$ (Eqn. 1)

$F_{t}=b_{0}+\left( 1+b_{1} \right).S_{t}+b_{2}.F_{t-1}+ b_{3}. S_{t-1}+ b_{4}. F_{t-2}+ \delta_{t}$ (Eqn. 2)

where *ε_t_* and *δ_t_* are normally distributed errors with mean 0 and variances *σ^2^_ε_* and *σ^2^_δ_* respectively.

The objective of the simulations was to produce vole densities that captured the observed short-term temporal structure of vole fluctuations and the long term variations, i.e. with realistic variation in spring densities while autumn vole densities remained approximately constant. Cornulier *et al.* (2013) showed that these long-term patterns of change appeared to result essentially from changes in winter population growth rates *a_0_*, estimated from -1.54 to -3.47 in Kielder data for the high-amplitude (1985-1998) and the low-amplitude period (1999-2010) respectively. We used the parameter values estimated by Cornulier et al. (2013) to simulate vole abundance time series from equations 1 and 2 (see above). Trial and error adjustments indicated that the match between the distributions of observed and simulated data was improved by adding 0.2 to the standard deviations *σ_ε_* and *σ_δ_* and by using -1.90 for *a_0_* instead of -1.54 (Table S3). We incremented winter growth rates by step of 0.1 from -1.90 so as to encompass the observed vole densities in spring (over 18 growth rates). The season-specific vole densities predicted by the model are compared to observed values in Figure S3.

Reference

Cornulier T, Yoccoz NG, Bretagnolle V, *et al.* (2013) Europe-wide dampening of population cycles in keystone herbivores. *Science*, **340**, 63–66.

Table S3. Parameters used for vole time series simulations (Equations 1 and 2), with values as estimated by Cornulier et al. (2013). Asterisks show values subsequently adjusted to improve the distribution of simulated values (see above text).

|  | Period 1  (1985-1998) | Period 2  (1999-2010) |
| --- | --- | --- |
| *a_1_* | -0.226 | -0.630 |
| *a_2_* | -0.121 | -0.148 |
| *a_3_* | -0.076 | 0.012 |
| *a_4_* | -0.006 | 0.020 |
| *b_1_* | -0.655 | -0.531 |
| *b_2_* | -0.096 | -0.173 |
| *b_3_* | -0.473 | -0.241 |
| *b_4_* | 0.306 | 0.073 |
| *a_0_* | -1.536* | -3.470 |
| *b_0_* | -3.765 | -3.571 |
| *σ_ε_* | 0.371* | 0.371* |
| *σ_δ_* | 0.549* | 0.549* |

Figure S3: Comparison of observed and simulated vole time series. Triangles, circles and diamonds indicate observed mean vole density from the high-amplitude period (1985-1998, in green), the low-amplitude period (1999-2010, in red) and for the whole period respectively (in black), in spring and autumn. Grey crosses show the range of simulated vole densities produced by the increments in winter population growth rate.

**Appendix S4. Functional response of clutch size of tawny owls to spring vole density and wNAO.**

We analysed the functional response of clutch size (for breeding pairs, range: 1-6 eggs) to spring vole density and wNAO. As for the number of fledglings, we applied a multinomial distribution. Model selection procedure identified the additive combination of vole density (log) and wNAO as the most parsimonious model (Table S4.1). Coefficients and their standard errors are presented in Table S4.2. The effect size related to wNAO was similar for clutch size and number of fledglings (Figure S4.1 & S4.2). This result indicates that most of the reduction in brood size at fledging associated with wNAO arose from a reduction in clutch size, rather than from increased chick mortality.

Table S4.1: Selection of models investigating the functional response of clutch size (N = 977) to vole density and winter climate. The number of parameters (*np*), the AICc difference between the current model and the most parsimonious one (*ΔAICc*), AICc weights (*wAICc*) and proportion of deviance explained (*R²*) are shown. The model with the strongest support is indicated in bold.

| Vital rate and model | *np* | *ΔAICc* | *wAICc* | *R²* | *Equation* |
| --- | --- | --- | --- | --- | --- |
| *Clutch size* |  |  |  |  |  |
| Null | 5 | 230.3 | 0 | - |  |
| log(Vole in spring) | 10 | 68.7 | 0 | 37.4 |  |
| wNAO | 10 | 208.1 | 0 | 7.0 |  |
| **log(Vole)+wNAO** | **15** | **0** | **0.70** | **54.6** | See Table S4.2 below |
| log(Vole)×wNAO | 20 | 1.7 | 0.30 | 56.5 |  |

Table S4.2: Coefficients and standard errors from the best multinomial model explaining variation in clutch size (log(Vole)+wNAO).

| Clutch size | Intercept | log(Vole in spring) | wNAO |
| --- | --- | --- | --- |
| *Coefficients* | |  |  |
| 2 | 3.786 | -0.329 | 0.075 |
| 3 | -2.739 | 1.224 | -0.110 |
| 4 | -10.841 | 2.782 | -0.322 |
| 5 | -19.278 | 4.083 | -0.563 |
| 6 | -48.664 | 9.322 | -0.167 |
| *Standard errors* | |  |  |
| 2 | 1.919 | 0.447 | 0.087 |
| 3 | 1.928 | 0.448 | 0.086 |
| 4 | 2.123 | 0.484 | 0.094 |
| 5 | 3.991 | 0.836 | 0.165 |
| 6 | 32.928 | 6.300 | 0.809 |

Figure S4.1: Comparison of the functional response of (a) clutch size and (b) number of fledglings to spring vole density and wNAO. Black and white symbols are for years with positive and negative wNAO index respectively. Lines are predicted values taken from the best model (solid and dashed lines for first and third quartiles of wNAO respectively).

Figure S4.2: Relative probabilities of cutch size according to spring vole density predicted by the best model (log(Vole)+wNAO) under (a) a low wNAO regime (1^st^ quartile, wNAO = -0.35) and (b) a high wNAO regime (3^rd^ quartile, wNAO = 2.76). The width of the grey area is proportional to the probability of observing a given clutch size and for a given vole density, the relative probability for each clutch size sums to one (see Appendix S2 for comparative purpose with number of fledglings).

**Appendix S5. Confidence intervals for the simulated number of territorial female tawny owls in Kielder Forest according to varying mean vole density in spring.**

Figure S5: Projected quantiles of the number of territorial females after 250 years according to spring vole densities as simulated by auto-regressive models (coloured lines with symbols: median; solid grey lines: 25-75%; dashed grey lines: 2.5-97.5%). Panels show simulations performed under positive (top panel, red triangles up), intermediate (middle panel, green circles) and negative (blue, triangle down) wNAO regimes. Each point differs by 0.1 unit of vole winter population growth.

**Appendix S6. Simulated dynamics of non-territorial tawny owls (floaters) according to varying vole dynamics.**

Figure S6: Evolution of the number of floaters over the first 50 years of simulations (N = 5000 iterations). Initial population vector was N_T_ = 100, N_F_ = 20 and N_J_ = 75. Green, yellow and red lines are median projections from simulated vole dynamics mimicking high-amplitude cycle period, average and low-amplitude periods respectively (see Figure 5).
